# Out to sea: ocean currents and patterns of asymmetric gene flow in an intertidal fish species

**DOI:** 10.3389/fgene.2023.1206543

**Published:** 2023-06-28

**Authors:** Anthony A. Snead, Andrey Tatarenkov, John C. Avise, D. Scott Taylor, Bruce J. Turner, Kristine Marson, Ryan L. Earley

**Affiliations:** ^1^ Department of Biological Sciences, University of Alabama, Tuscaloosa, AL, United States; ^2^ Department of Ecology and Evolutionary Biology, University of California, Irvine, Irvine, CA, United States; ^3^ Brevard County EEL Program, Melbourne, FL, United States; ^4^Department of Biological Sciences, Virginia Tech, Blacksburg, VA, United States

**Keywords:** gene flow, ocean current, population structure, Kryptolebias marmoratus, mangrove forest, biophysical modeling, Lagrangian (Lagrangian parcel tracking)

## Abstract

Passive dispersal via wind or ocean currents can drive asymmetric gene flow, which influences patterns of genetic variation and the capacity of populations to evolve in response to environmental change. The mangrove rivulus fish (*Kryptolebias marmoratus*), hereafter “rivulus,” is an intertidal fish species restricted to the highly fragmented New World mangrove forests of Central America, the Caribbean, the Bahamas, and Florida. Mangrove patches are biological islands with dramatic differences in both abiotic and biotic conditions compared to adjacent habitat. Over 1,000 individual rivulus across 17 populations throughout its range were genotyped at 32 highly polymorphic microsatellites. Range-wide population genetic structure was evaluated with five complementary approaches that found eight distinct population clusters. However, an analysis of molecular variance indicated significant population genetic structure among regions, populations within regions, sampling locations within populations, and individuals within sampling locations, indicating that rivulus has both broad- and fine-scale genetic differentiation. Integrating range-wide genetic data with biophysical modeling based on 10 years of ocean current data showed that ocean currents and the distance between populations over water drive gene flow patterns on broad scales. Directional migration estimates suggested some significant asymmetries in gene flow that also were mediated by ocean currents and distance. Specifically, populations in the center of the range (Florida Keys) were identified as sinks that received migrants (and alleles) from other populations but failed to export individuals. These populations thus harbor genetic variation, perhaps even from extirpated populations across the range, but ocean currents and complex arrangements of landmasses might prevent the distribution of that genetic variation elsewhere. Hence, the inherent asymmetry of ocean currents shown to impact both genetic differentiation and directional migration rates may be responsible for the complex distribution of genetic variation across the range and observed patterns of metapopulation structure.

## 1 Introduction

Gene flow, the exchange of genetic material between populations, can drive spatial patterns of genetic variation (Slatkin, 1985; Slatkin, 1987), which in turn influences the rates at which genetic drift and natural selection alter allele frequencies (Lenormand, 2002; Gandon and Nuismer, 2009; Blanquart, Gandon, and Nuismer, 2012; Greenbaum et al., 2014; Tamagawa et al., 2022). Gene flow can maintain genetic variation in declining populations experiencing strong genetic drift by introducing alleles from adjacent populations (Epps et al., 2005; Gompert et al., 2021). While gene flow generally decreases the impact of genetic drift, it can increase or decrease rates of local adaptation (North et al., 2011; Frankham, 2015; Tigano and Friesen, 2016). In populations with reduced standing genetic variation, increased gene flow can introduce adaptive alleles from adjacent populations (Caprio and Tabashnik, 1992; Aitken and Whitlock, 2013; Bontrager and Angert, 2019). Such introductions could facilitate genetic rescue or increased population growth rates caused by more than just the demographic contributions of immigrants, namely, the arrival of high fitness genotypes (Frankham, 2015; Whiteley et al., 2015). Conversely, high gene flow between populations in which strong selection is operating can homogenize allele frequencies, attenuate rates of local adaptation (Aeschbacher et al., 2017; Cornwell, 2020; Micheletti and Storfer, 2020), and reduce species-level genetic variation through the loss of private alleles (Baillie et al., 2016). Gene flow also decreases genetic differentiation between populations, thereby maintaining species’ integrity (i.e., preventing speciation; Kisel and Barraclough, 2010; Slatkin, 1985; Slatkin, 1987). Patterns of gene flow are often driven by the distance between populations (Wright, 1943; Aguillon et al., 2017) and the environmental conditions in the intervening area (Schwartz et al., 2009; Thomas et al., 2015). Therefore, identifying and determining how factors that influence gene flow might affect patterns of genetic differentiation among populations is essential to understanding current evolutionary dynamics and predicting species’ responses to future environmental change.

Patterns of gene flow in species that actively disperse are often symmetrical (Alexander et al., 2019; DiLeo et al., 2013; Munshi-South, 2012; but see Corrigan et al., 2016; Waterhouse et al., 2018). Species with passive dispersal, however, can experience asymmetric gene flow driven by winds (Kling and Ackerly, 2021) or water currents (Xuereb et al., 2018; Fisher et al., 2022). Passive dispersal can reduce species’ resilience to climate change by restricting their ability to actively shift their range in response to environmental challenges (Kling and Ackerly, 2021). Asymmetric gene flow can result in heterogenous patterns of genetic diversity (Sjöqvist et al., 2015; Rougemont et al., 2021) that impact phenotypic variation within populations and subsequently determine whether populations can adapt to local conditions (Baltazar-Soares and Eizaguirre, 2016). Furthermore, asymmetric gene flow can govern opportunities for genetic rescue (Matz et al., 2018; Waterhouse et al., 2018) because, for instance, potentially adaptive alleles might not be introduced to maladapted populations. Hence, explicitly testing for landscape-wide patterns of directional gene flow can provide insights into the distribution of alleles and genetic differentiation between populations.

Mangrove forests are globally threatened coastal ecosystems (Blanco-Libreros and Estrada-Urrea, 2015) dominated by salt-tolerant woody trees (i.e., mangroves). Mangrove trees are foundation species (Osland et al., 2013; Osland et al., 2017), dominant species that drive community structure through primarily non-trophic interactions such as providing structural support and modulating nutrient flow (Dayton, 1972; Ellison et al., 2005; Ellison, 2019). Mangroves mediate abiotic conditions within the forest through alterations in the microclimate, sediment accretion, and organic soil content (Guo et al., 2017), while hosting unique biotic communities (Laegdsgaard and Johnson, 1995; Huxham et al., 2004) compared to adjacent habitats. For example, Laegdsgaard and Johnson (1995) found that the fish community within Australian mangrove forests was younger and more diverse with 27 additional species exclusively in Australian mangrove forests compared to nearby seagrass beds. Mangrove patches thus can be considered biological islands separated by an intervening matrix of inhospitable habitats that can influence patterns of gene flow between patches (Bergemann et al., 2009; Lin et al., 2020; Zheng et al., 2021). Ocean currents offer passive transport through the intervening matrix between populations and often dictate gene flow patterns between resident populations of mollusks (Ratsimbazafy and Kochzius, 2018), crabs (Britto et al., 2018), isopods (Baratti et al., 2011), and mangrove trees themselves (Ngeve et al., 2016). Here, we explore whether and how the distance between mangrove patches and ocean currents interact to predict observed patterns of genetic differentiation among populations of a small euryhaline killifish, *Kryptolebias marmoratus* (Costa, 2004), hereafter “rivulus.”

Rivulus is endemic to mangrove forests from Central America to Central Florida, the Bahamas, and the Caribbean (Taylor, 2012; Tatarenkov et al., 2017). They are tolerant of a wide range of environmental conditions including high levels of hydrogen sulfide (Abel, Koenig, and Davis, 1987; Rey et al., 1992; Taylor, 2012), low levels of dissolved oxygen (Dunson and Dunson, 1999; Turko et al., 2014), temperatures from 7°C to 38°C (Taylor et al., 1995), and salinities from 0 to 80 parts per thousand (ppt) (Kristensen, 1970; Taylor et al., 1995; Taylor, 2000). Even with a large geographic range and broad environmental tolerances, rivulus’ distribution is patchy owing to some combination of anthropogenic activity, biotic interactions, dispersal limitations, and abiotic conditions (Snead and Earley, 2022). In fact, rivulus populations often are characterized by low genetic diversity and low levels of gene flow that cannot be explained by distance (Tatarenkov et al., 2007; Tatarenkov et al., 2012; Avise and Tatarenkov, 2015; Tatarenkov et al., 2017). While rivulus dispersal is not well understood, passive dispersal of embryos or adults rafting on/in flotsam via ocean currents or extreme weather events (e.g., hurricanes) has been proposed (Tatarenkov et al., 2012). Indeed, rivulus often attach their eggs to floating debris (Mourabit et al., 2011) and are found within moist rotting logs that could be swept out to other locations (Taylor et al., 2008). Because rivulus populations are androdiecious, composed primarily of self-fertilizing hermaphrodites and few males (Harrington. 1971; Mackiewicz et al., 2006), a single fertilized egg or hermaphroditic individual could colonize new habitats. Therefore, the observed distribution of rivulus might be due to limited dispersal driven by ocean currents that, combined with low genetic diversity within populations, restrict rivulus’ response to climate change through range shifts, genetic rescue, or evolutionary rescue.

We hypothesized that rivulus would have strong population structure across its range accompanied by genetic differentiation between populations, as has been demonstrated in prior studies (Tatarenkov et al., 2007; Tatarenkov et al., 2012; Avise and Tatarenkov, 2015; Tatarenkov et al., 2017). The mechanisms responsible for observed patterns of genetic differentiation, however, remain unknown. We thus hypothesized that ocean current mediated dispersal (oceanic connectivity) and the distance between populations over water would predict patterns of population genetic differentiation. We predicted that increased oceanic connectivity would be negatively associated with population genetic differentiation by promoting gene flow, while increased distance would be positively associated with genetic differentiation by impeding gene flow. Finally, we hypothesized that gene flow is asymmetric and driven by oceanic connectivity. Specifically, we predicted that increased oceanic connectivity would be positively associated with asymmetric gene flow estimates. By combining biophysical models and population genetics, we quantify how ocean currents mediate symmetric and asymmetric gene flow in an intertidal species that occupies patches restricted by adjacent terrestrial and marine habitats.

## 2 Materials and methods

### 2.1 Samples

We used 1,245 genetic samples consisting of previously published (Tatarenkov et al., 2007; Tatarenkov et al., 2012; Avise and Tatarenkov, 2015; Tatarenkov et al., 2017) and unpublished data collected between 1994 and 2014 across 56 sites. From each fish, fin clips were taken and stored in either 95% ethanol or salt saturated DMSO before extracting genomic DNA using a proteinase K method described in Tatarenkov et al. (2012). For each sample, we amplified 32 unlinked microsatellite markers (Mackiewicz et al., 2006) using multiplex reactions, described in Tatarenkov et al. (2012). After alleles were separated in a GA 3100 instrument, they were scored in Genemapper 4.0 (Applied Biosystems) and binned following Tatarenkov et al. (2010).

### 2.2 Spatial analysis and biophysical modeling

**FIGURE 1:**
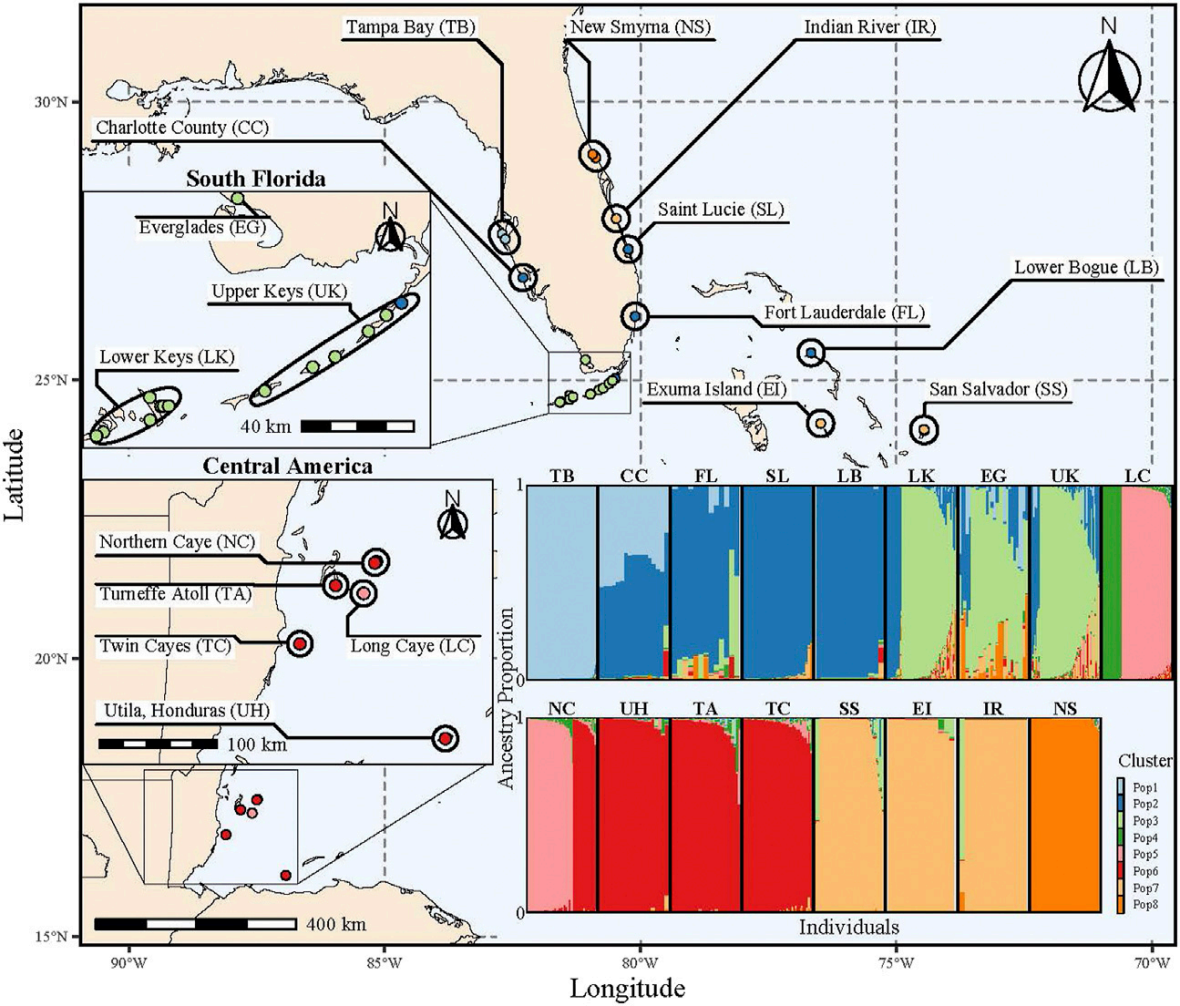
Map highlighting all sampling locations and their population groups (ellipses). Each point is colored by the majority ancestry assignment determined by STRUCTURE using K = 8; colors correspond to the ancestry bar plot (bottom right). The ancestry bar plot shows the proportion of ancestry for a K = 8 on the y-axis for each individual (x-axis) faceted by population grouping. The width of the individual bars vary to make the population groupings equal in width for visual purposes; therefore, the population groups do not have the same number of samples in each group.

A 10 km buffer was placed around each sampling location using the package *sf* (Pebesma, 2018) to match the resolution of the coarsest Ocean General Circulation Model (OGCM; ∼9 km). Overlapping buffers were grouped into populations resulting in 20 total populations from Honduras to North Central Florida. Individuals with more than 10% missing genotypic data (i.e., more than 6 missing alleles) were removed, and populations with less than 10 individuals were removed reducing the number of populations to 17 and number of individuals to 1,120 (Figure 1; Supplementary Table S1). While the genetic samples were collected across a broad time interval, previous research found samples collected from the same population over a decade apart had low genetic differentiation (F_ST_ = 0.023; Tatarenkov et al., 2007). Similarly, in the Florida Keys, previous work demonstrated genetic differentiation between time points within a population was low (F_ST_ = 0.002) and genetic differentiation was nonsignificant (Tatarenkov et al., 2012). The distance between populations over water henceforth ‘water distance’ was calculated by masking landmasses, rasterizing the marine habitat using a value of one, and calculating the least cost path between population centroids with the R package *gdistance* (Etten, 2017) and *rnaturalearth* (Massicotte and South, 2023).

To estimate oceanic connectivity, we used Connectivity Modeling System (CMS; Paris et al., 2013) to track particles spatiotemporally in a simulated ocean current environment; here, particles represent embryos or rafting adults. At its simplest configuration, CMS is a deterministic model in that particles released from the same location at the same time will follow identical trajectories. By designating a horizontal diffusivity coefficient, a random component can be added to the motion of each particle at each timestep (Okubo, 1971). The horizontal diffusivity coefficient is scaled by the timestep and grid size to account for the sub-grid turbulent processes not resolved by the OGCM (Okubo, 1971). Ocean current data were downloaded from the Hybrid Coordinate Ocean Model (HYCOM) GLBu0.08 expt_19.1 and GOMu.0.04 expt_50.1 with a uniform 0.08° and 0.04° grid size, respectively, on a 3-h output frequency (Chassignet et al., 2007). Thus, we used 36.5 (GLBu0.08 expt_19.1) and 12.3 m^2^s^-1^ (GOMu.0.04 expt_50.1) as horizontal diffusivity coefficients within the turbulent diffusion model of CMS (equation 3 in Paris et al., 2013) following equation four in Okubo (1971). We selected 1,000 random locations (with replacement) that were an additional 2 km offshore (12 km from the nearest sampling location) along the seaward edges of the population buffers to ensure that all particles entered the simulation without being washed ashore during the first timestep. Ten particles were released from each location daily between the years 2000 and 2010 (10 particles *x* 1,000 locations *x* 17 population groupings *x* 365 days *x* 10 years = 620.5 million particles) at random times with the location of each particle retained every half hour. Particles were tracked for 63 days, which is the average time rivulus spend in the egg before successfully hatching (33 days) plus the twice the standard deviation (15 days; Earley and Marson unpublished data). A 63-day tracking period was chosen so that the simulations include 97.5% of the observed variation in successful hatching time. Because eggs or adults in flotsam that wash ashore can be exported again with ebbing tides, particles could not become stranded when encountering land masses.

From the resulting particle trajectory files, the amount of time any particle spent within the receiving population’s 10 km buffer (Time Spent In Buffer; TSIB) was calculated and summed for each source—sink combination. Some particles were only inside the population buffer at one time point, potentially due to the time interval of sampling across the simulation or ocean currents pushed the particle rapidly through the buffer. In either case, not incorporating those particles would misrepresent connectivity; therefore, any particle with only one time point was assigned a half hour of residence which was the time interval of sampling. Because oceanic connectivity is inherently asymmetric (*Pop*_*1*_ *→ Pop*_*2*_ *≠ Pop*_*2*_ *→ Pop*_*1*_), estimates were retained as asymmetric values for directional migration analyses and made symmetric for analyses with genetic differentiation estimates. For the symmetric dataset, TSIB was summed per population pair regardless of direction (*TSIB*_*Pop1 → Pop2*_ *+ TSIB*_*Pop2 →Pop1*_). While the summed TSIB is referred to as symmetric, the values include natural occurring asymmetries in ocean currents because the values are summed by population pair not averaged. Hence, the symmetric dataset is mathematical symmetric but still includes asymmetric processes that maybe evolutionarily relevant. However, direct connectivity via ocean currents did not exist for every population pair or in both directions; therefore, TSIB for each population pair was divided by the total TSIB across all population pairs and used as the relative probability of successful dispersal. The relative probability of successful dispersal was used to construct an undirected and directed graph (i.e., network) in the package *igraph* (Csardi and Nepusz, 2006) for the symmetric and asymmetric datasets, respectively; populations were the nodes and relative probability of successful dispersal were the edges. The package *igraph* was used to find the most direct path with highest relative dispersal probability; however, *igraph* finds the shortest path by summing all potential pathways between two nodes and retaining the shortest. Therefore, all edges (i.e., relative probabilities) were natural log transformed and multiplied by −1 before using *igraph* to find the shortest path. The transformation produces positive edge values with high relative dispersal success having smaller numbers (i.e., shorter distances). The exponential of the shortest path multiplied by −1 was divided by the number of nodes (i.e., intermediate populations) to get the per simulation relative probability of dispersal based on time. Summing transformed relative probabilities and taking the exponential of the sum multiplied by −1 is equivalent to multiplying two relative probabilities. Hence, resulting values indicate the relative probability of the most direct path scaled by the number of intermediate nodes.

### 2.3 Genetic analyses

All subsequent analyses were completed within R version 4.2.2 (R Core Team, 2022). Loci were tested for Hardy-Weinberg equilibrium (HWE) across the entire dataset and per population using the package *pegas* (Paradis, 2010). While all loci departed from HWE in at least one population, none did so in all populations; therefore, all loci were retained for analysis. Mean observed heterozygosity, expected heterozygosity, and mean allelic richness were calculated with the packages *heirfstat* (Goudet and Jombart, 2022) and *PopGenReport* (Adamack and Gruber, 2014). To evaluate the distribution of genetic variance across the range, populations were grouped into one of five regions (West Florida: TB and CC; South Florida: EG, LK, UK and FL; East Florida: SL, IR, and NS; Bahamas: SS, EI, and LB; Central America: NC, LC, TC, TA, and UH; Figure 1), and an Analysis of MOlecular Variance (AMOVA) was implemented in the package *poppr* (Kamvar et al., 2014) with four hierarchal levels (region, population, sampling location, individuals) and significance was tested with 999 permutations (Excoffier et al., 1992) using *ade4* (Bougeard and Dray, 2018).

Population structure was evaluated to explore the spatial patterns of genetic dissimilarity using complementary methods. We performed a Discriminant Analysis of Principal Components (DAPC; Jombart et al., 2010) using the package *adegenet* (Jombart and Ahmed, 2011) retaining all principal component axes and evaluating K values of 1 through 60. Results from the DAPC (Supplementary Figure SA1) identified a rapid decrease in the Bayesian Information Criterion (BIC) for K = 1 through K = 5 and appeared to asymptote near K = 30; therefore, we evaluated K = 5 through K = 30 in four complementary ways: 1) TESS3 with 20 replicates and a maximum of 100,000 iterations using the package *tess3r* (Caye et al., 2016); 2) sNMF with 20 replicates, 100,000 iterations, and an alpha of 100 using the package *LEA* (Frichot and Francois, 2015); 3) STRUCTURE with 200,000 iterations, 50,000 iteration burn-in, and default parameters for three chains (Pritchard, Stephens, and Donnelly, 2000); and 4) InStruct for 100,000 iterations and 50,000 burn-in iterations for three chains (Gao, Williamson, and Bustamante, 2007). For TESS3 and sNMF, potential K values were identified by examining the minimum cross entropy value for each K. For STRUCTURE, we implemented the Evanno method (Evanno et al., 2005) and compared the loglikelihood of the data. While neither the Evanno method (Evanno et al., 2005) nor the loglikelihood of the data have been validated for use with InStruct, we implemented them in InStruct for comparison with STRUCTURE, but also evaluated the Deviance Information Criterion (DIC; Spiegelhalter et al., 2002).

Pairwise population differentiation was evaluated with G_ST_ (Nei and Chesser, 1983), G′_ST_ (Hedrick, 2005) and Jost’s D (Jost, 2008) using the package *mmod* (Winter 2012). R_ST_ (Slatkin, 1995) was also calculated between populations using the package *polysat* (Clark et al., 2011). G_ST_ (Nei and Chesser, 1983) was chosen for comparison with other metrics and previous studies; however, G′_ST_ (Hedrick, 2005) and Jost’s D (Jost, 2008) were specifically chosen for use with highly polymorphic microsatellite data. G_ST_ assumes bi-allelic loci and is based on heterozygosity (Nei and Chesser, 1983); therefore, the maximum G_ST_ decreases as population genetic diversity increases and systematically underestimates genetic distance (Hedrick, 1999). G′_ST_ attempts to account for this downward bias by standardizing G_ST_ (Nei and Chesser, 1983) by scaling the observed G_ST_ by its theoretical maximum given the mean within-population heterozygosity (Hedrick, 2005). Jost’s D estimates genetic differentiation using allelic diversity rather than heterozygosity (Jost, 2008). R_ST_ was chosen for comparison as it still suffers from the biases of G_ST_; however, R_ST_ includes the mutational distance between microsattelite markers (Slatkin, 1995). We estimated directional migration with BayesAss version 3 (Wilson and Rannala, 2003) for 30 million iterations, a 10 million iteration burn-in, and thinning interval of 10,000 for five chains using 0.25, 0.085, and 0.18 for the mixing parameters for allele frequencies (Δ_A_), migration rates (Δ_M_), and inbreeding coefficients (Δ_F_) respectively. For all Bayesian analyses (STRUCTURE, InStruct, BayesAss), convergence was assessed with Tracer v1.7.1 (Rambaut et al., 2018) and the scale reduction factor (Brooks and Gelman, 1998).

To evaluate isolation-by-distance and isolation-by-oceanography patterns, all genetic differentiation estimates were linearized (G_ST_/(1—G_ST_)) and water distance log10-transformed before downstream analyses (Rousset, 1997). For normality, we took the square root of relative probability of successful dispersal via ocean currents based on TSIB, which will be referred to as OC_S_ and OC_A_ for symmetric and asymmetric estimates, respectively. Mantel and partial Mantel tests were run between all linearized genetic differentiation estimates, water distance, and OC_S_ after controlling for water distance in the package *ecodist* (Goslee and Urban, 2007). To evaluate asymmetric patterns of gene flow, Mantel and partial Mantel tests were run between directional migration estimates (BayesAss), water distance, and OC_A_ after controlling for water distance; here, we developed and used a custom Mantel and partial Mantel function based on code from *ecodist* (Goslee and Urban, 2007). The custom tests do not require symmetric matrices because they transform matrices into vectors and use those vectors to derive the null distribution used to calculate the *p*-value. While most packages require symmetric matrices, the test does not inherently require symmetry (Mantel, 1967). Because Mantel and partial Mantel tests can be underpowered (Legendre and Fortin, 2010; Guillot and Rousset, 2013; Legendre et al., 2015; Zeller et al., 2016), we also used a Maximum-Likelihood Population Effects model (MPLE; Clarke et al., 2002) implemented in *nlme* (Pinheiro et al., 2023) with *cormple* (Pope, 2022). MLPE models account for non-independence between pairwise comparisons by incorporating the covariance between pairwise comparisons that share a common population as a random effect to account for the non-independence of pairwise data (Clarke et al., 2002). Models were run for each combination of water distance, OC (OC_S_ & OC_A_), and their two-way interaction as predictor variables (scaled and centered) against each linearized genetic differentiation metric (e.g., G′_ST_) and the fourth root of the migration rate, transformed for normality. Models then were compared using AICc following Burnham and Anderson 2004.

## 3 Results

### 3.1 Genetic diversity

Mean observed heterozygosity, expected heterozygosity, and allelic richness ranged from 0 to 0.52, 0.07 to 0.69, and 1.37 to 6.13 respectively (Supplementary Table S2). While population in Central America (e.g., TA, TC) had the highest observed heterozygosity, expected heterozygosity, and allelic richness, the Florida Key (i.e., UK and LK) has comparably high levels of genetic diversity that is considerably higher that population in the Bahamas and the rest of Florida.

### 3.2 Population structure

The AMOVA identified significant population differentiation at each level (*p* = 0.001) with >10% of the variance attributed to each level (Table 1). Given that the best value of K depends on the biological question (Meirmans, 2015; Janes et al., 2017), multiple K values were tested for each clustering algorithm (Supplementary Table S3; Supplementary Figure S1). STRUCTURE, using the Evanno method, identified K = 8 as the most likely number of clusters (Supplementary Table S3; Supplementary Figure S1). While the remaining cluster algorithms (i.e., InStruct, TESS3, sNMF) all identified different values of K as the most likely, they all either had inflection points (TESS3 & sNMF) or identified (InStruct with Evanno method) K∼8 as a likely number of clusters indicating that K∼8 is useful for interpreting broad scale population structure. While K = 5 was untestable by the Evanno method, the DAPC plot does indicate broadscale differentiation between Florida and Central America (Axis 1), South Florida and New Smyrna (Axis 2), and South Florida and West Florida (Axis 3; Supplementary Figure S2).

**TABLE 1:** Percent variance attributed to each hierarchal level of the AMOVA along with ϕ and the *p*-value.

Level	Variance (%)	ϕ	*p*-value
Among Regions	18.72	0.19	0.001
Among Populations within a Region	11.45	0.14	0.001
Among Locations within a Population	19.91	0.29	0.001
Among Individuals within a Location	37.46	0.75	0.001
Within Individuals	12.46	0.88	0.001

### 3.3 Genetic differentiation and migration

**FIGURE 2:**
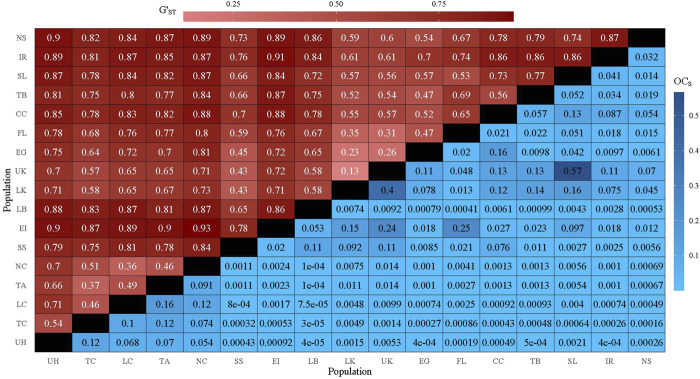
Heatmap displaying pairwise G′_ST_ (upper triangle) and OC_S_ (square-root transformed; lower triangle). Diagonal denoted by black tiles.

Pairwise G_ST_, G′_ST_, Jost’s D, and R_ST_ ranged from 0.03 to 0.69, 0.132 to 0.927, 0.08 to 0.817, and 0.03 to 0.66, respectively (Figure 2; Supplementary Figures S3, S4). Regardless of genetic distance metric, the Upper Keys (UK) and Lower Keys (LK) were most genetically similar (G_ST_ = 0.03, G′_ST_ = 0.13, D = 0.08, R_ST_ = 0.03; Figure 2; Supplementary Figures S3, S4).With G′_ST_ and Jost’s D, Exuma Island (EI) and Northern Caye (NC) were most genetically dissimilar (G′_ST_ = 0.93, D = 0.82; Figure 2; Supplementary Figure S3), while the most genetically distinct populations measured with G_ST_ were Exuma Island (EI) and Indian River (IR) (G_ST_ = 0.69; Supplementary Figure S2). With R_ST_, Exuma Island (EI) and New Smyrna (NS) were the most genetically distinct (R_ST_ = 0.66; Supplementary Figure S4). Excluding estimated retention rates (0.67—0.96), directional migration estimated with BayesAss (Wilson and Rannala, 2003) ranged from 0.001 to 0.175 with the lowest migration rate going from Long Caye (LC) to Exuma Island (EI; 0.001) and the highest migration rate from Everglades (EG) to Lower Keys (LK; 0.175; Supplementary Figure S5). Directional migration rate confidence intervals were calculated according to Wilson and Rannala 2003 (1.96 x standard deviation). If confidence intervals for migration estimates in both directions did not overlap zero or each other, the population pair was considered to exhibit asymmetric gene flow (Supplementary Figure S5).

### 3.4 Symmetric gene flow

Mantel tests between pairwise genetic distance metrics and water distance identified significant isolation by distance with each metric (*p* < 0.02; Table 2), while partial Mantel tests between genetic distance and OC_S_ after controlling for water distance were either marginally non-significant (G_ST_ & G′_ST_), nonsignificant (Jost’s D) with 0.08 < *p* < 0.15, or significant (R_ST_; Table 2). After comparing models with AICc, the most likely MLPE model for each genetic distance metric included OC_S_ (Table 3). However, all G_ST_ models and the top three R_ST_ models were equally likely, being within 2 AICc values (Burnham and Anderson, 2004; Table 3). For both G′_ST_ and Jost’s D, the full model (Genetic Distance ∼ OC_S_ + Distance + OC_S_ *x* Distance) was best with an AICc_w_ > 0.99 and separated from the next best model by >10 AICc units (Table 3). For both G′_ST_ and Jost’s D, all variables in the best model (Distance, OC_S_, and their interaction) were significant (*p* < 0.03); both OC_S_ and the interaction between OC_S_ and water distance were significantly negatively associated with genetic distance, while water distance was significantly positively associated with genetic distance (Table 3).

**TABLE 2:** Mantel and partial Mantel test results testing the correlation between genetic distances (G_ST_, G′_ST_, Jost’s D, R_ST_), water distance, and oceanic connectivity (OC) using the r package *ecodist* (top) and our custom function (bottom).

ecodist	G_ST_			G′_ST_			D			R_ST_	
Formula	Value		*p*-value	Value		*p*-value	Value		*p*-value	Value	*p*-value
∼ Distance	0.13 (0.02, 0.32)		**0.018**	0.5 (0.43, 0.59)		**<0.0001**	0.68 (0.66, 0.72)		**<0.0001**	0.28 (0.16, 0.44)	**<0.0001**
∼ OC_S_ + Distance	−0.15 (−0.22, −0.06)		0.086	−0.15 (−0.22, −0.08)		0.091	−0.12 (−0.18, −0.05)		0.137	−0.18 (−0.26, −0.09)	**0.042**

*Custom*	G_ST_			G′_ST_			D			R_ST_			MR		
Formula	Value		*p*-value	Value		*p*-value	Value		*p*-value	Value	*p*-value		Value		*p*-value
∼ Distance	0.13 (0.07, 0.33)		0.056	0.5 (0.46, 0.62)		**<0.0001**	0.68 (0.66, 0.75)		**<0.0001**	0.68 (0.66, 0.75)	0.002		−0.23 (−0.28, −0.09)		**0.0002**
∼ OC* + Distance	−0.15 (−0.2, −0.02)		**0.016**	−0.15 (−0.19, −0.05)		**0.025**	−0.12 (−0.16, −0.03)		0.066	−0.18 (−0.22, −0.06)	0.005		0.06 (0.02, 0.17)		0.146

The formula is on the left with the Mantel statistics (value), the 95% confidence interval located under the statistic in the format (lower 95%, upper 95%), and the one-sided *p*-value with significance indicated in bold text. OC_S_ and OC_A_ refer to the symmetric and asymmetric oceanic connectivity estimate, respectively, and MR refers to the migration rate estimated by BayesAss. The asterisk (*) indicates that OC in the lower table containing the results from the custom Mantel and partial Mantel tests is OC_S_ for evaluation against genetic distances (G_ST_, G′_ST_, and Jost’s D) and OC_A_ for analysis with BayesAss migration rates.

**TABLE 3:** Results from maximum likelihood population effect (MLPE) models for each genetic distance metric (G_ST_, G′_ST_, Jost’s D and R_ST_), and migration rate (MR) with AICc, AICc_w_, coefficients (Beta, β), 95% confidence intervals, and their *p*-value with significance indicated in bold text.

						OC			Distance				OC x distance		
Formula	AICc			AICcw		β		*p*-value	Β			*p*-value	β		*p*-value
G_ST_ ∼ OC_S_	−71.96			0.33		−0.04 (−0.07, −0.01)		**0.014**							
G_ST_ ∼ OC_S_ + Distance	−71.55			0.25		−0.03 (−0.06, 0.01)		0.14	0.02 (−0.01, 0.05)			0.212			
G_ST_ ∼ Distance	−71.32			0.24					0.03 (0, 0.06)			**0.019**			
G_ST_ ∼ OC_S_ + Distance + OC_S_ *x* Distance	−70.96			0.17		−0.04 (−0.09, 0)		0.06	0.02 (−0.01, 0.05)			0.204	−0.02 (−0.06, 0.02)		0.24
G′_ST_ ∼ OC_S_ + Distance + OC_S_ *x* Distance	453.24			>0.99		−0.51 (−0.83, −0.19)		**0.002**	1.04 (0.83, 1.24)			**< 0.0001**	−0.58 (−0.85, −0.31)		**< 0.0001**
G′_ST_ ∼ Distance	466.59			0					1.05 (0.86, 1.24)			**< 0.0001**			
G′_ST_ ∼ OC_S_ + Distance	468.65			0		−0.04 (−0.29, −0.21)		0.75	1.03 (0.81, 1.25)			**< 0.0001**			
G′_ST_ ∼ OC_S_	530.77			0		−0.66 (−0.94, −0.38)		**< 0.0001**							
D ∼ OC_S_ + Distance + OC_S_ *x* Distance	162.63			>0.99		−0.13 (−0.24, −0.02)		**0.025**	0.52 (0.45, 0.6)			**< 0.0001**	−0.19 (−0.29, −0.1)		**0.0001**
D ∼ Distance	174.45			0					0.51 (0.44, 0.58)			**< 0.0001**			
D ∼ OC_S_ + Distance	176.26			0		0.03 (−0.06, 0.12)		0.555	0.52 (0.44, 0.6)			**< 0.0001**			
D ∼ OC_S_	282.72			0		−0.29 (−0.41, 0.17)		**< 0.0001**							
RST ∼ OCS + Distance + OCS x Distance	−31.3			0.46		−0.06 (−0.12, 0)		0.023	0.06 (0.02, 0.09)			**0.002**	−0.04 (−0.09, 0)		0.07
RST ∼ Distance	−30.25			0.27					0.07 (0.04, 1)			**< 0.0001**			
RST ∼ OCS + Distance	−30.18			0.26		−0.03 (−0.07, 0.01)		0.154	0.06 (.02, 0.09)			**0.003**			
RST ∼ OCS	−23.55			0		−0.06 (−0.1, −0.03)		**< 0.0001**							
MR ∼ OC_A_ + Distance + OC_A_ *x* Distance	−822.99			0.86		0 (−0.01, 0.01)		0.479	−0.01 (−0.02, 0)			**0.005**	−0.01 (−0.02, 0)		0.009
MR ∼ OC_A_ + Distance	−818.28			0.08		0.01 (0, 0.01)		0.09	−0.01 (−0.02, 0)			**0.007**			
MR ∼ Distance	−817.57			0.06					−0.01 (−0.02, −0.01)			**0.0003**			
MR ∼ OC_A_	−812.89			0.005		0.01 (0, 0.02)		**0.003**							

### 3.5 Asymmetric gene flow

For comparison with past landscape and seascape genetic research, we developed a custom Mantel test that does not require symmetric matrices. To test our custom function (Detailed in the *Mantel—Asymmetric* section of the supplied code; See Data Availability Statement below), we evaluated the function on symmetric data and compared the results with *ecodist*. Our function and *ecodist* produced the same correlation values but the custom function identified OC_S_ as significant in the partial Mantel tests with G_ST_ (*p* = 0.016), G′_ST_ (*p* = 0.025) and R_ST_ (*p* = 0.005) but marginally non-significant with Jost’s D (*p* = 0.066; Table 2), while *ecodist* identified OC_S_ as marginally non-significant (G_ST_: *p* = 0.085; G′_ST_: *p* = 0.092), not significant (D: *p* = 0.146) or significant (R_ST_: *p* = 0.042). While our custom function and *ecodist* calculate significance similarly, our function first converts the matrix into a vector and permutes the vector rather than matrix thereby changing, compared to existing software, the permutation procedure on which the null distribution is generated. Our custom function identified a significant negative Mantel’s r (r = −0.23; *p* = 0.00002) between water distance and migration rate and a nonsignificant positive association between migration rates and OC_A_ after controlling for water distance (*p* = 0.146). The full MLPE model (Migration Rate ∼ OC_A_ + Distance + OC_A_ *x* Distance) was the most likely model and ∼4.6 AICc units lower than the second-best model (Migration Rate = OC_A_ + Distance) and ∼5.4 AICc lower than the model with water distance alone with an AICc_w_ > 0.86 (Table 3). In the best model, water distance (*p* = 0.005) and the interaction between water distance and OC_A_ (*p* = 0.009) were significantly negatively associated with migration rate, while OC_A_ alone was nonsignificant (*p* = 0.479; Table 3).

## 4 Discussion

Passive dispersal strategies limit the ability of a species to dictate their dispersal trajectory, thereby hindering range shifts in response to environmental change (Kling and Ackerly, 2020) while impacting the distribution of genetic variation through complex patterns of gene flow (Nikula et al., 2013; Rougemont et al., 2021). Passive dispersal strategies often depend on wind or ocean currents that are inherently asymmetric (Kling and Ackerly, 2020), resulting in directional patterns of gene flow that can dictate the potential for local adaptation through genetic rescue (Matz et al., 2018; Kling and Ackerly, 2021; Rougemont et al., 2021). There is a growing body of literature demonstrating the importance of long-distance, passive ocean current-mediated gene flow in coastal and marine systems (Schunter et al., 2011; Damerau et al., 2012; Xuereb et al., 2018), but our study is the first to evaluate how ocean currents affect genetic structure in an intertidal fish species while also explicitly quantifying relationships between asymmetric gene flow and surface currents. By combining genetic data from over a thousand individuals from populations across rivulus’ range with biophysical models based on 10 years of ocean current simulations, we find evidence that ocean currents and distance over water may drive patterns of gene flow and asymmetric migration rates.

### 4.1 Population structure

Range-wide population structure was assessed using four complementary methods (STRUCTURE, InStruct, TESS3, sNMF) with 1,120 fin clips genotyped at 32 highly polymorphic microsatellite markers collected from across rivulus’ range. With coarse resolution (K = 8), methods were partially congruent, showing overlapping clusters and assignments (Supplementary Figure S6); however, there were notable discrepancies between methods (Supplementary Figure S6). The discrepancies between clustering methods indicate the importance of comparing across methodologies before interpreting ancestry results. For example, STRUCTURE clusters the southern Bahamian populations of San Salvador and Exuma Island with Indian River located on the east coast of Central Florida (Figure 1; Supplementary Figure S6). However, all other methodologies cluster Indian River with the Florida Keys (LK and UK; Supplementary Figure S6), and G_ST_ identified Exuma Island and Indian River to be the most genetically dissimilar (Supplementary Figure S3). Neither of these clusters were expected because neither the Florida Keys nor Southern Bahamian populations are nearest to Indian River so, interpreting STRUCTURE results without auxiliary support could lead to inappropriate inferences, which highlights the importance of conducting complementary analyses.

At K = 8, patterns of population structure and ancestry generally recapitulate previous work (Tatarenkov et al., 2017); however, our increased sample size and focus on rivulus, as opposed to the *K. marmoratus* species complex that includes *K. hermaphroditus*, simultaneously identified large blocks of shared ancestry, novel patterns of admixture (e.g., TB → CC ← FL), and distinct population clusters (e.g., NS; Figure 1). Clustering analysis generally grouped nearby populations together but there were some notable exceptions. Populations within the Florida Keys and Florida Bay (EG, LK, and UK) cluster together with limited, albeit the most, admixture from an adjacent distinct cluster containing both CC and SL, which are populations located on the west and east coast of Florida. Therefore, Florida Keys and Florida Bay populations are genetically distinct from the nearest population groups, located to the northeast and northwest. In Central America, Utila Island (Honduras), Turneffe Atoll (Belize), and Twin Cayes (Belize) formed a cluster that shows admixture with Northern Caye (Belize). Northern Caye was split between the Utila Island, Turneffe Atoll, and Twin Cayes cluster and Long Caye (Belize). Even though Long Caye is near the other Central American populations, Long Caye shares significant ancestry only with Northern Caye, and Long Caye harbors a unique genetic cluster found within an inland freshwater pond that is disconnected from seawater (Figure 1; Turko et al., 2018; Turko et al., 2022). Colonization of less saline environments can result in rapid population divergence in marine fish (Beheregaray and Sunnucks, 2001; Kelly et al., 2006) and salinity can be a strong source of selection (Velotta et al., 2022). Hence, even at regional scales, we identify a genetically distinct cluster that could potentially be maintained by local adaptation.

While our discussion is focused on broad scale clustering results (K = 8), an AMOVA suggests significant population structure between regions (Central America, Bahamas, South Florida, West Florida, East Florida), populations within regions, sampling locations within populations, individuals within sampling locations, and within individuals (Table 1). Strong population structure is common in intertidal fish (Díez-del-Molino et al., 2016; Hurt et al., 2017; Xie et al., 2022) and species occupying highly fragmented landscapes (Browne et al., 2015; Wultsch et al., 2016). We also expect strong population structure in mixed-mating species (Mori et al., 2015), those that can outcross or self-fertilize, with more structure emerging as outcrossing rates decrease (Duminil, et al., 2007; Hodgins and Yeaman, 2019). As primarily self-fertilizing hermaphrodites, a single rivulus individual or fertilized egg could colonize a new habitat fragment, resulting in extreme genetic homogeneity. If a newly founded population is isolated for an extended period due to habitat fragmentation or restricted gene flow, that population might locally adapt and outcompete immigrants, which should reduce successful gene flow from adjacent populations even if dispersal occurs (Tobler et al., 2009; Logan et al., 2016; Barbraud and Delord, 2021). Interestingly, the northernmost rivulus populations, Tampa Bay and New Smyrna, show the least mixed ancestry across the range and the most distinct clustering even at K = 8, suggesting that the populations might have arisen through rare founder events followed by limited gene flow and local adaptation. Our range-wide assessment of rivulus population structure adds more evidence to support the role of fragmentation, and mating system in driving population structure.

### 4.2 Gene flow

Previous work did not detect significant isolation by distance; however, previous work focused on local or regional scales while the current study focused on the entire species range (Tatarenkov et al., 2012; Tatarenkov et al., 2015). Both our Mantel tests and maximum likelihood population effects models (MLPE) identify distance over water as a significant predictor of genetic differentiation (Table 2; Table 3). This demonstrates that the spatial configuration of populations and distribution of landmasses are important drivers of population structure. The MLPE models also identified oceanic connectivity (OC_S_) and its interaction with water distance as important predictors of genetic differentiation (Table 3). Ocean current mediated gene flow often is attributed to physical boundaries (Truelove et al., 2017; Xuereb et al., 2018); however, in this study, the distance between populations is calculated based on marine habitat thereby including physical boundaries in the distance measure. Therefore, the significance of ocean currents in this study is not due to the additional distance caused by circumventing landmasses because the additional distance is already accounted for within our distance measure. Furthermore, our network approach enabled the estimation of indirect oceanic connectivity between populations allowing for a steppingstone pattern whereby two populations may only be connected via ocean currents through intermediate populations. By dividing our estimate of indirect connectivity by the number of intermediate nodes, we standardized our connectivity estimate by the steps required to connect two populations (Supplementary Figure S6). Importantly, OC_S_ includes asymmetric ocean current patterns; therefore, the results suggest that unidirectional ocean current driven connectivity may impact genetic differentiation between rivulus populations. Ocean currents influence patterns of genetic differentiation in other species (Truelove et al., 2017; Huyghe and Kochzius, 2018; Xuereb et al., 2018), but these species often have pelagic larvae that can respond to oceanic stimuli to trigger settlement or vertical migration (Kelly and Palumbi, 2010; Díaz-Ferguson et al., 2012). Adult rivulus and eggs rafting on floating debris, however, cannot guide their own long-distance dispersal success, relying completely on surface currents to wash migrants ashore. Unlike previous studies that quantified the number of particles exchanged between populations (Truelove et al., 2017; Xuereb et al., 2018), we quantified the amount of time a particle spent within the population buffer. This approach enabled us to concurrently evaluate how ocean currents affect exchange of particles between populations and the impact of local ocean currents surrounding the receiving population on particle retention. While previous work demonstrates that local ocean surface currents have strong impacts on the retention of propagules by the source population (Teske et al., 2015; Feng et al., 2016), research has not explicitly evaluated the potential for local ocean surface currents surrounding the receiving population to promote dispersal by retaining immigrants. Hence, our results suggest that studies should expand from quantifying only the number of particles exchanged between two locations to include potential impacts of local surface currents that can drive the probability of successfully settling in a new location.

**FIGURE 3:**
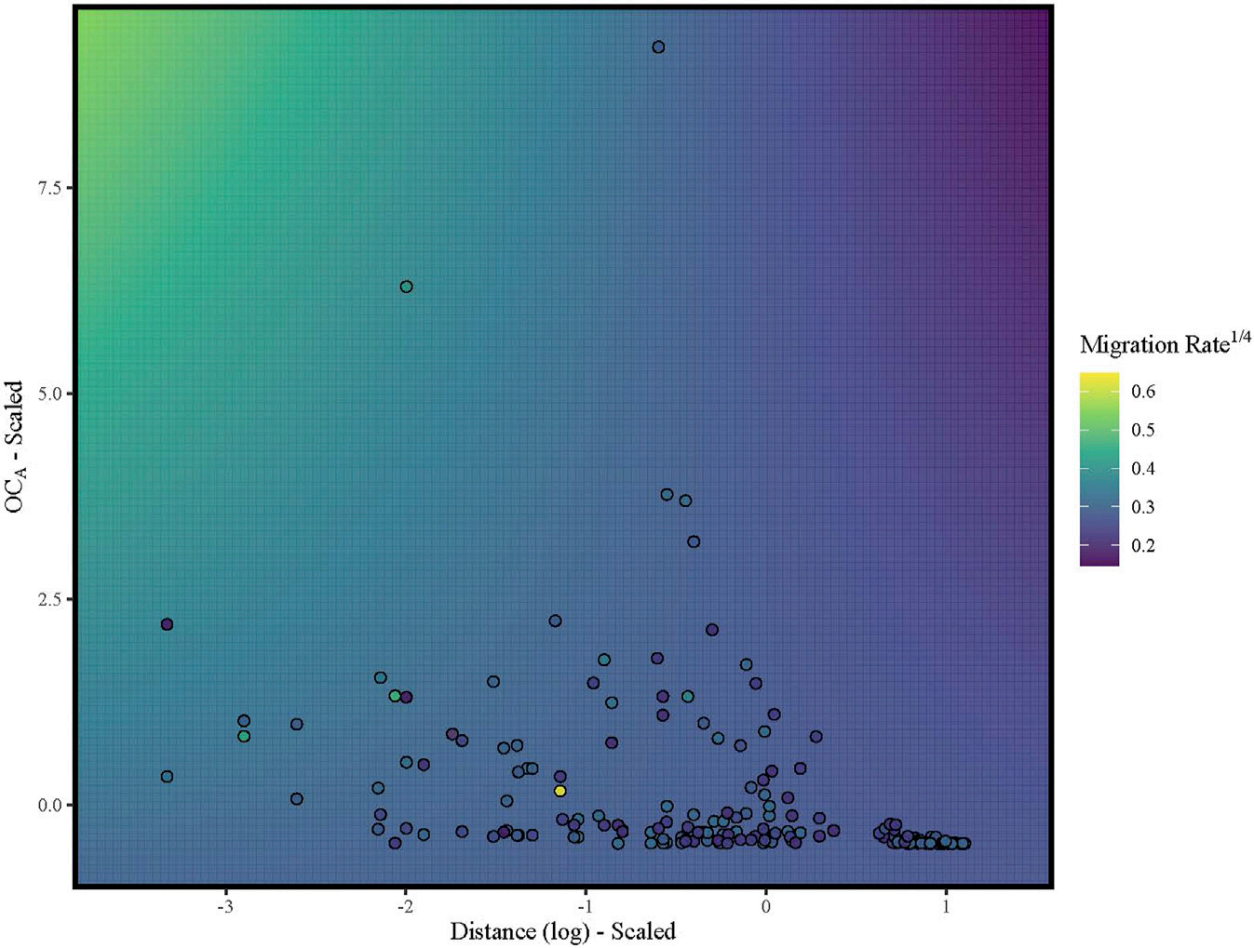
Heatmap illustrating the association between asymmetric migration rates (fourth rooted for normality) and the interaction term (log(distance) x OC_A_) with both water distance and OC_A_ (scaled and centered). Points are pairwise rates of asymmetrical migration colored according to the legend (purple = low migration rates; yellow = high migration rates). Background color represents predicted migration rates from a linear model to visualize the interaction term.

While asymmetric ocean currents impact the extent to which populations are genetically differentiated (Teske et al., 2015; Xuereb et al., 2018), quantifying genetic differentiation alone fails to identify asymmetries in gene flow which can impact the future spread of potentially adaptive alleles, which would then limit opportunities for genetic rescue (Matz et al., 2018; Waterhouse et al., 2018). Ocean currents are inherently directional, and the direction of currents may change over time, for instance, seasonally or over evolutionary time scales (Seigel et al., 2008; Buffoni et al., 2015). Therefore, we explicitly tested whether directional ocean current connectivity predicts directional migration rates. Our MLPE models suggested that the full model with oceanic connectivity, water distance, and their interaction was the best model even though only water distance and its interaction with ocean currents were statistically significant (Table 3). While the coefficient of the interaction term was negative (Table 3), increases in oceanic connectivity (OC_A_) were positively associated with migration rates when the distance between populations over water was low to moderate (Figure 3). Our measure of ocean current connectivity did not account for the time that particles spend out to sea before arriving at the receiving population, and rivulus eggs are likely not equally viable across the duration considered. In other species, egg hatching probability decreases as eggs age (Dahlberg, 1979) and environmental conditions during egg development can impact hatching success and the speed of development (Tubbs et al., 2005; Bobe and Labbé, 2010). Rivulus has greater hatching success at lower salinities (McCain et al., 2020), and eggs exposed to air early in development can be subsequently triggered to hatch by exposure to aerial hypoxia (Wells et al., 2015). Furthermore, our biophysical model released a uniform number of particles each day across the simulation; however, rivulus exhibit reproductive seasonality that may vary across regions (Lomax et al., 2017). Hence, the timing and number of potential dispersers might show spatiotemporal variability that influences the probability of dispersal via ocean current. Therefore, even though our integration of biophysical modeling with genetic estimates of directional migration identified ocean currents as an important driver, differential egg viability and reproductive seasonality may have increased the error surrounding our oceanic connectivity estimates and highlighted potential avenues for future work focused on directional migration in marine and intertidal species.

While distance over water and its interaction with asymmetric ocean current connectivity were significant predictors of directional migration rates, migration estimates were generally small (median <0.008) with large confidence intervals (Supplementary Figure S5). Ocean surface currents are highly stochastic because local weather conditions and marine variables (Seigel et al., 2008; Buffoni et al., 2015) can change the magnitude and direction of the current across both short and long timescales (hours to millennia; Liu and Weisberg, 2005; Robinson, 1975; Sawada and Handa, 1998). Therefore, the large confidence intervals surrounding our per generation migration rates could be the product of ocean current variability along with rivulus’ short generation time (∼100 days; Cole and Noakes, 1997). Even low rates of directional migration can have large consequences for the spatial distribution of genetic variation and the spread of potentially adaptive alleles across the landscape (Rice and Papadopoulos, 2009; Sundqvist et al., 2016). Therefore, stochastic bouts of asymmetric migration may be a property of this system, resulting in periods of isolation interspersed with large influxes of migrants.

We found low rates of directional migration overall, but high migration rates (>0.15) were detected from Everglades (EG) and Fort Lauderdale (FL) to Florida Keys (UK and LK). Importantly, such migration was not reciprocated; that is, EG and FL did not receive many migrants from Florida Keys (Supplementary Figure S5). Indeed, Florida Keys received immigrants from populations across the range but, export from Florida Keys is an order of magnitude lower if it occurs at all (Supplementary Figure S5). These data indicate that islands in the Florida Keys are sinks despite being centrally located in the range. The Florida Keys harbor high genetic diversity and allelic richness relative to the rest of the range (Supplementary Table S2), with significant population structure even within that localized area (Tatarenkov et al., 2012). Migrant retention within Florida Keys may be due to a combination of ocean currents and the spatial distribution of landmasses. Surface currents from all directions converge on the Florida Keys, which could enable rafting adults or eggs to colonize (Lee et al., 1992; Weisberg et al., 2019). The Florida Keys also have landmasses distributed throughout the seascape in staggered fashion, which could facilitate retention of dispersers. Hence, local topography and more fine scale oceanographic processes are likely influencing dispersal across rivulus’ range, enabling certain locations to retain migrants more effectively while limiting their export. In this case, the Florida Keys might preserve genetic variation from previously extirpated populations, but this variation could become trapped in the Florida Keys unless ocean currents change. While not incorporated within this study, new methodologies are being developed for coastal areas that could provide more resolution into the role of local ocean current patterns on passive dispersal success in high complex intertidal systems (Gotoh and Khayyer, 2018; Roberts et al., 2019). Unfortunately, predicting how ocean currents will change with climate comes with a significant level of uncertainty (Hirschi et al., 2020; Hu et al., 2020; Bellomo et al., 2021). Even with these methodological limitations, asymmetries in migration identified potential genetic sinks. These genetic sinks could both facilitate (via elevated genetic and perhaps phenotypic variation) local adaptation to future environmental change within the sinks and interrupt the spread of potentially adaptive alleles through the range, limiting the potential for genetic rescue.

### 4.3 Conclusion

Using 10 years of biophysical simulations, we found that ocean currents are important predictors of both genetic differentiation and asymmetric gene flow, shedding light on potential drivers of rivulus’ relatively chaotic distribution of genetic variation and demonstrating that ocean currents can modulate gene flow in an intertidal species with internal fertilization and no pelagic larvae. However, the coarse resolution of current OGCM used within our biophysical models likely missed interactions between surface currents, topography, and landmass distributions that might further influence retention and export of migrants and, as a result, metapopulation structure and the spread of genetic variation across the landscape. We suggest that the Florida Keys, although central to the range, act as a genetic sink that retains migrants but does not export individuals to other populations. This could potentially be due to fine scale oceanographic processes such as eddies or tides that are currently difficult to incorporate in biophysical models. Coincidentally, new ocean modeling approaches utilizing unstructured meshes are being developed that may offer greater resolution into hydrological dynamics in coastal and intertidal systems (Sein et al., 2017). Increased resolution and incorporation of age-dependent egg viability, differential egg survival due to environmental conditions, and phenology within the biophysical model may help to further explore the roles of local ocean current fluctuations and the ecology of rivulus in mediating genetic divergence and directional gene flow between populations. Our results illuminate a complex pattern of population structure likely driven by ocean currents that modulate directional but passive dispersal of rivulus across patchy mangrove forests.

## Data Availability

The datasets presented in this study can be fund in online repositories. The names of the repositories can be found below: https://github.com/anthonysnead/Rivulus_Ocean_Current_Gene_Flow_2023; https://figshare.com/s/d6cc089c98bee611aadb.
