# Supplementary material for: Out to sea: ocean currents and patterns of asymmetric gene flow in an intertidal fish species
Source: Front Genet. 2023 Jun 28;14:1206543. doi: 10.3389/fgene.2023.1206543 (PMC10349204; doi:10.3389/fgene.2023.1206543)
Supplement: Supplementary file 1 [file Table1.DOCX]

Supplementary Material

**Out to Sea: Ocean Currents and Patterns of Asymmetric Gene Flow in an Intertidal Fish Species**

**Anthony A. Snead^*^, Andrey Tatarenkov, John C Avise, D. Scott Taylor, Bruce J Turner, Kristine Marson, Ryan L Earley**

*** Correspondence:** Anthony A. Snead: [anthonysneadjr@gmail.com](mailto:anthonysneadjr@gmail.com)

# Supplementary Tables

**Supplementary Table 1** A table filled with the number of genetic samples collected for each population per year of collection.

| Population | 1994 | 1996 | 1997 | 2001 | 2005 | 2007 | 2009 | 2010 | 2011 | 2012 | 2013 | 2014 | Total |
| --- | --- | --- | --- | --- | --- | --- | --- | --- | --- | --- | --- | --- | --- |
| CC | 17 |  |  |  |  |  |  |  |  |  |  |  | 17 |
| EG |  |  |  |  | 28 |  |  |  |  |  |  |  | 28 |
| EI |  |  | 12 |  |  |  |  |  |  |  |  |  | 12 |
| FL |  |  |  |  |  |  |  |  |  |  |  | 13 | 13 |
| IR |  |  |  |  |  |  |  |  |  |  | 14 |  | 14 |
| LB |  |  |  |  |  |  |  |  |  |  | 14 |  | 14 |
| LC |  |  |  |  |  |  |  |  | 140 | 132 |  |  | 272 |
| LK |  |  |  |  |  | 40 |  | 53 | 23 | 27 |  |  | 143 |
| NC |  |  |  |  |  |  |  |  | 44 | 23 |  |  | 67 |
| NS |  |  |  |  |  |  |  |  |  |  | 92 |  | 92 |
| SL |  |  |  |  |  |  |  | 25 | 4 |  |  |  | 29 |
| SS |  |  |  | 4 |  |  |  |  |  |  |  | 77 | 81 |
| TA |  |  |  |  |  |  | 30 |  |  |  |  |  | 30 |
| TB |  |  |  |  |  |  |  |  |  | 130 |  |  | 130 |
| TC |  |  |  |  | 59 |  |  |  |  |  |  |  | 59 |
| UH |  | 20 |  |  |  |  |  |  |  |  |  |  | 20 |
| UK |  |  |  |  |  | 6 |  | 35 | 26 | 17 |  | 15 | 99 |
|  |  |  |  |  |  |  |  |  |  |  |  | **Total** | **1120** |

**Supplementary Table 2** The mean observed heterozygosity, expected heterozygosity, and allilic richness for each population used within the study.

| Population | Observed Heterozygosity | Expected Heterozygosity | Allelic Richness |
| --- | --- | --- | --- |
| CC | 0.02 | 0.26 | 1.92 |
| EG | 0.003 | 0.46 | 3.86 |
| EI | 0 | 0.15 | 1.37 |
| FL | 0 | 0.45 | 2.79 |
| IR | 0 | 0.07 | 1.45 |
| LB | 0.02 | 0.37 | 2.22 |
| LC | 0.16 | 0.6 | 4.59 |
| LK | 0.013 | 0.54 | 4.69 |
| NC | 0.2 | 0.6 | 4.1 |
| NS | 0.0009 | 0.18 | 1.93 |
| SL | 0.03 | 0.29 | 2 |
| SS | 0.005 | 0.39 | 3.05 |
| TA | 0.28 | 0.6 | 4.82 |
| TB | 0.0009 | 0.28 | 1.89 |
| TC | 0.52 | 0.69 | 6.13 |
| UH | 0.005 | 0.51 | 3.66 |
| UK | 0.05 | 0.56 | 5.15 |

**Supplementary Table 3** Results from the clustering algorithms split by the statistic used to evaluate them, mean log likelihood of the data or cross entropy. The most likely number of clusters interpreted from the statistic is indicated by the highest (STRUCTURE & InStruct) and lowest (TESS3 & sNMF) columns. For STRUCTURE and InStruct, the most likely and second most likely values of K identified by the Evanno method (ΔK) are provided while the inflection points, values where the downward trend of cross entropy ends, are provided for TESS3 and sNMF

| ***Mean Log likelihood of the Data – LnP(D)*** | | | |
| --- | --- | --- | --- |
|  | | Evanno (ΔK) | |
| Software | Highest | 1^st^ | 2^nd^ |
| STRUCTURE | 18 | 8 (12.617) | 19 (7.118) |
| InStruct | 5 | 20 (6.998) | 8 (6.406) |
|  |  |  |  |
| ***Cross Entropy*** |  |  |  |
| Software | Lowest | Inflection Points (↓ to ↑) | |
| TESS3 | 30 | 9, 12, 15, 28 | |
| sNMF | 26 | 12, 17, 19, 22, 26 | |

## Supplementary Figures

**
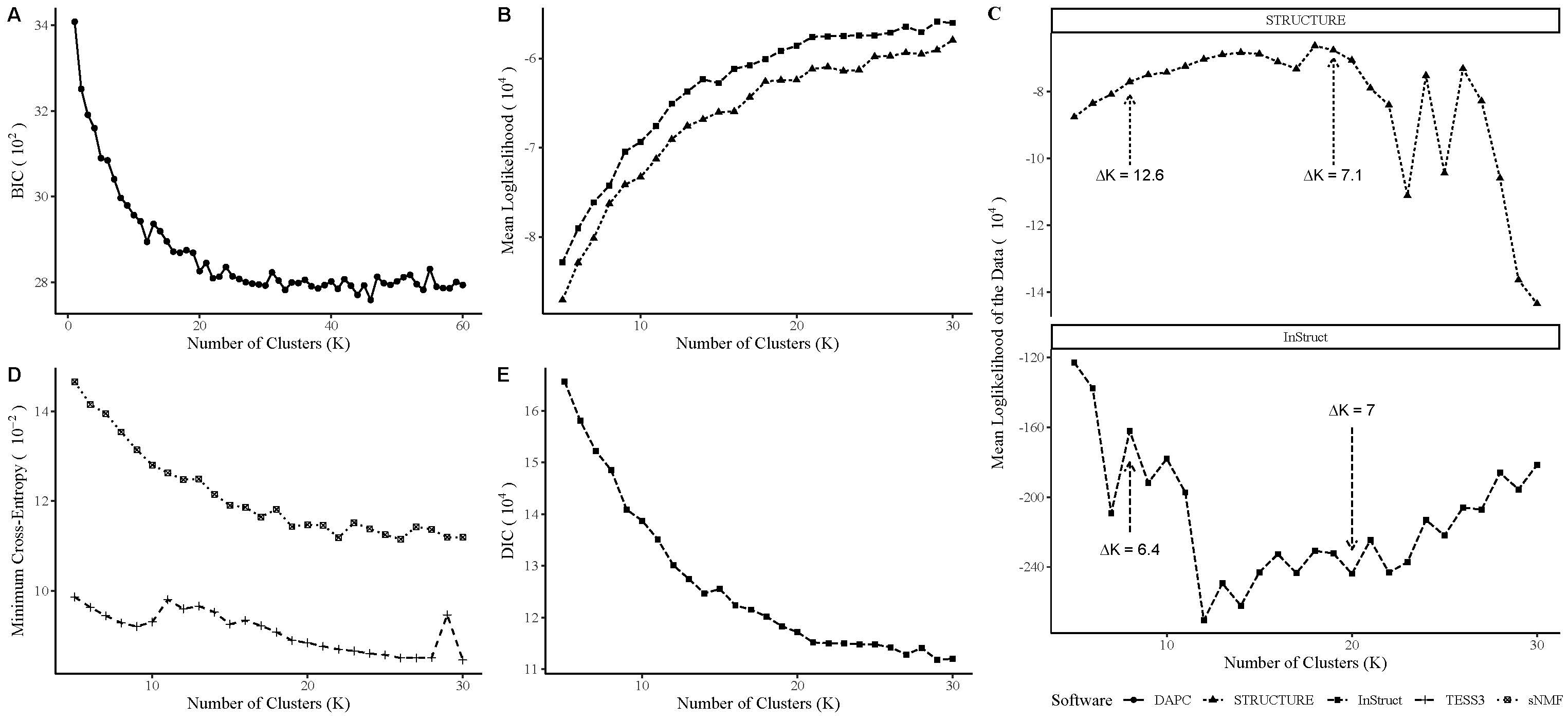
**

**Supplementary Figure 1:** Population structure clustering algorithm results from K=1 through K=60 (Panel A: DAPC) and K=5 through K=30 (Panels B – E; STRUCTURE, InStruct, TESS3, and sNMF) split into panels by the metric used for comparision (Panel A: BIC, Panel B: mean loglikelihood, Panel D: cross entropy, Panel E: DIC, and Panel C: mean loglikelihood of the data). Line type and point shape indicate the method following the legend at bottom right. ΔK refers to the rate of change used in the Evanno method, and the arrows highlight the two most likely values of K based on ΔK.

**
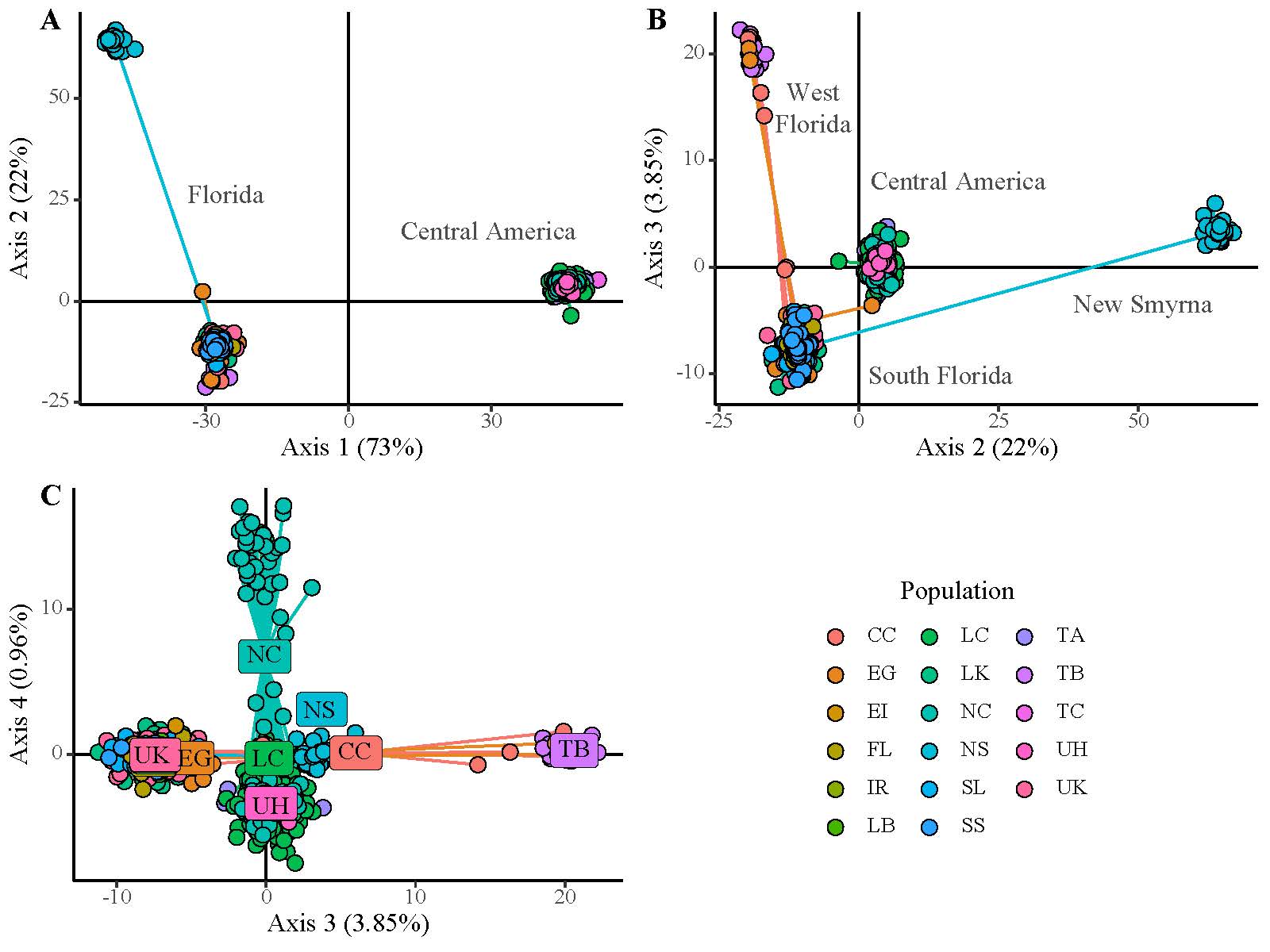
**

**Supplementary Figure 2** The Discriminant Analysis of Principal Components (DAPC) for K=8. Panel A visualizes Axis 1 and Axis 2. Panel B visualizes Axis 2 and Axis 3. Panel C visualizes Axis 3 and Axis 4. Either regions labels (Panels A and B) or representative population labels (Panel C) are included to aid in interpretation. Each point is an individual colored by the populations it belongs to.


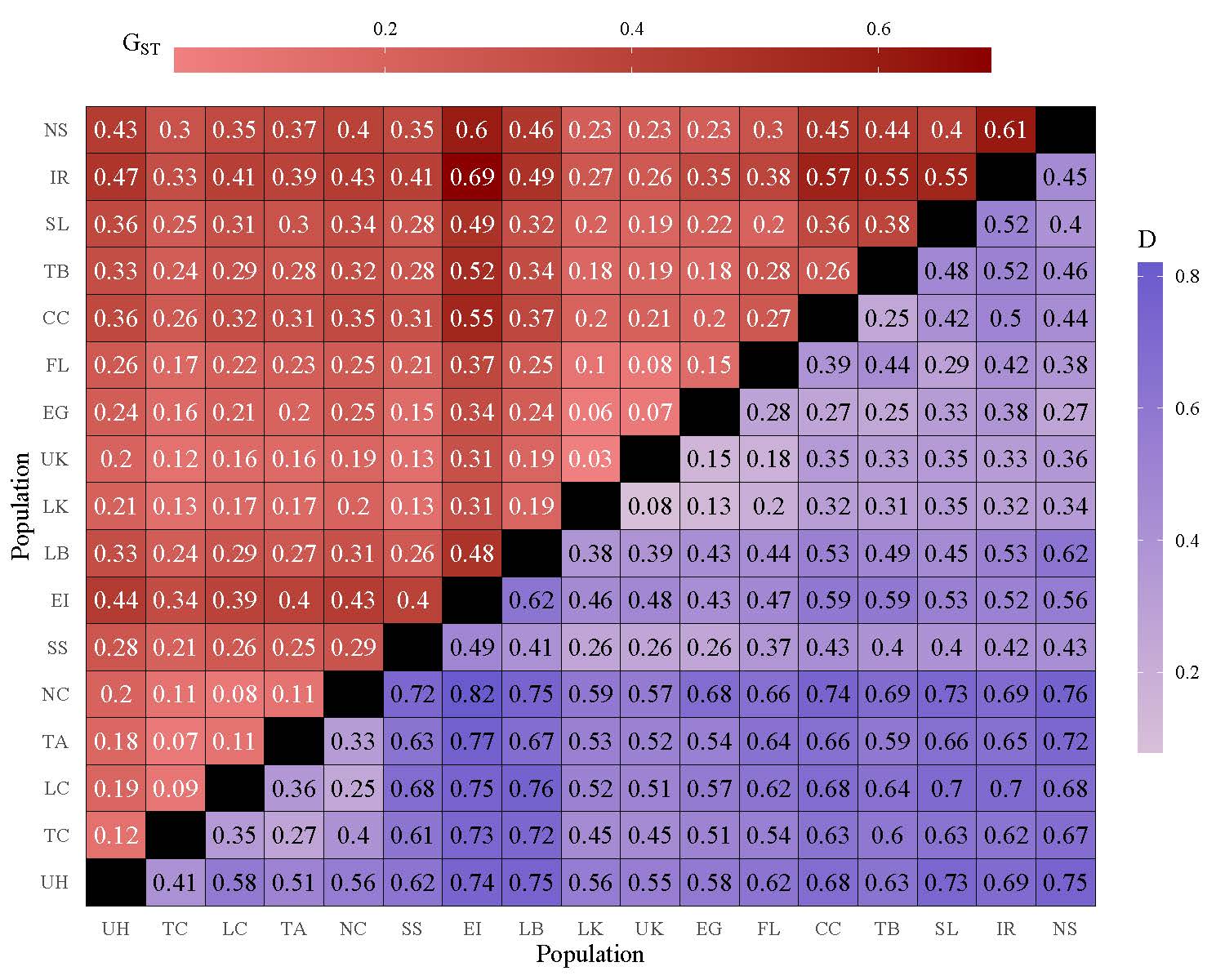


**Supplementary Figure 3:** Heatmap with population on the x- and y-axes filled with G_ST_ values in the upper triangle shaded in red and Jost’s D in the lower triangle shaded in purple.


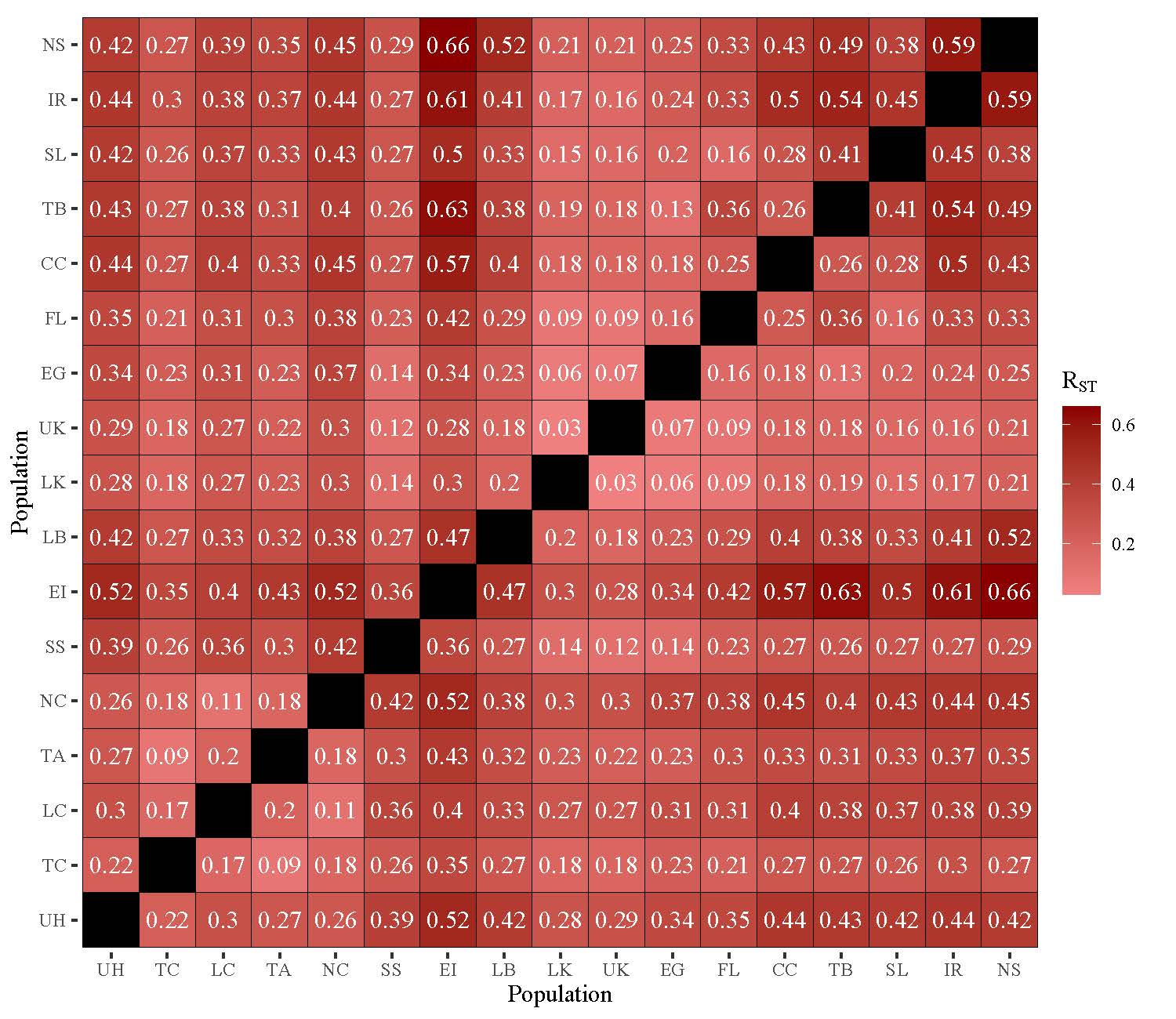


**Supplementary Figure 4** Heatmap with population on the x and y axes filled with R_ST_ values.


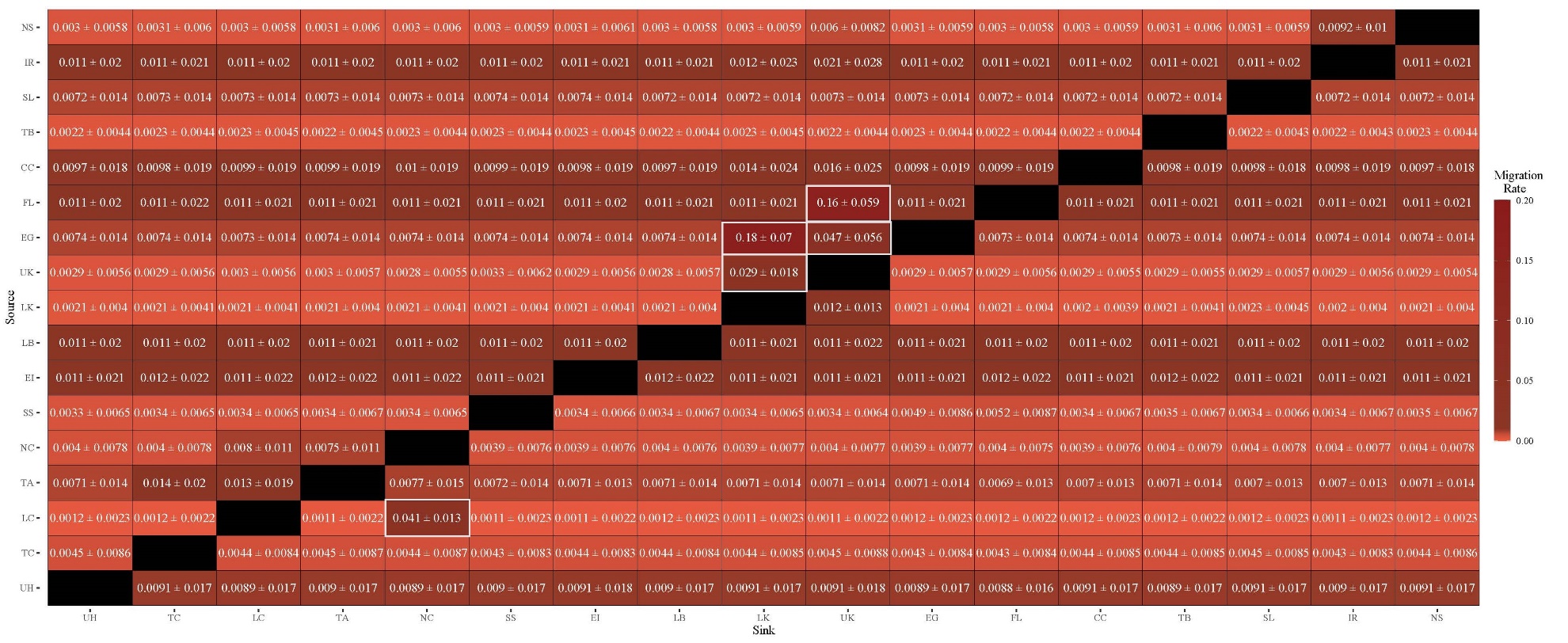
**Supplementary Figure 5:** Heatmap with source population on the y-axis and sink population on the x-axis filled with directional migration rates estimated by BayesAss with a rough 95% confidence interval (1.96 x standard deviation) and shaded in red. As you move across the row, each value is the estimated migration rate from the source to the sink. Particularly strong bouts of asymmetric migration are highlighted with white boxes.


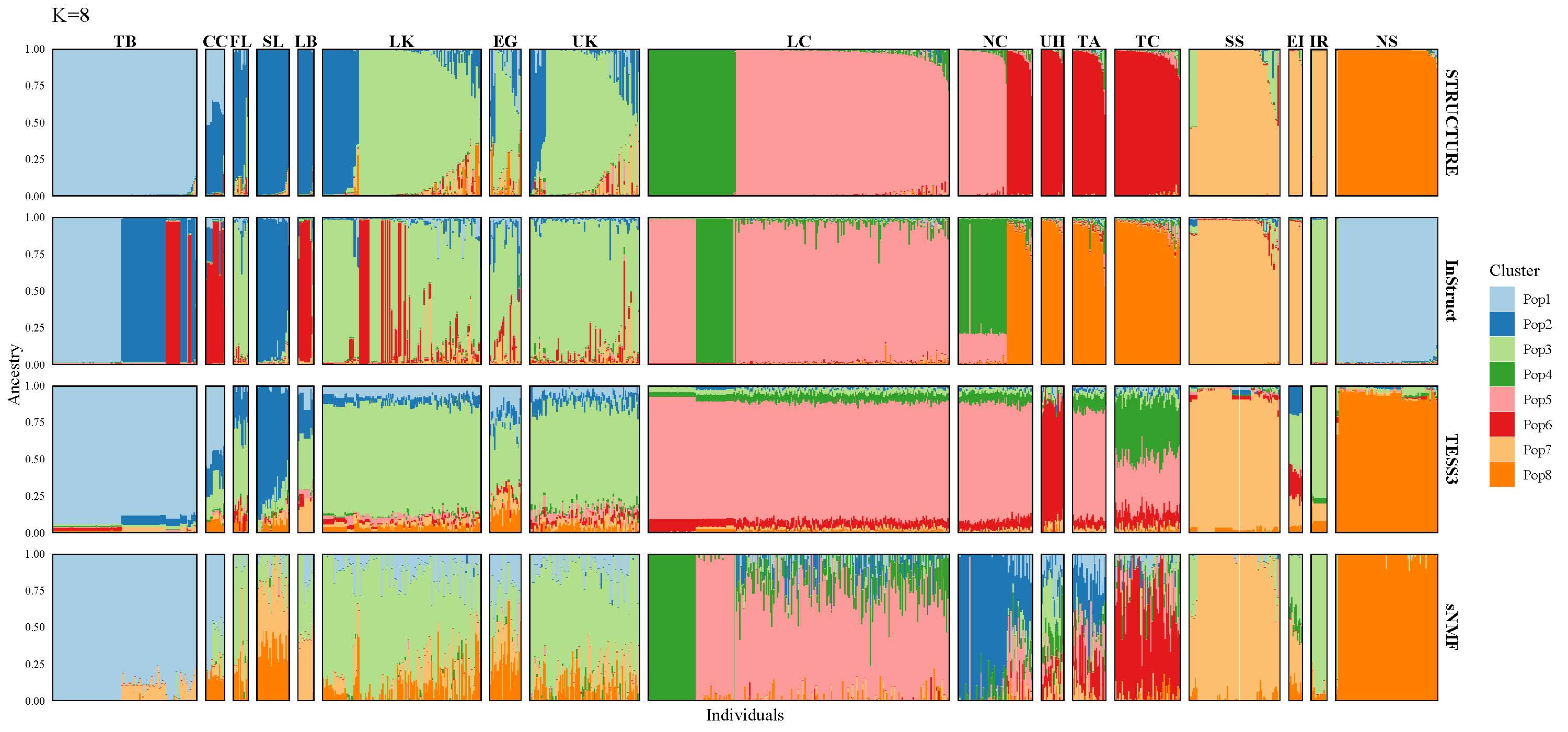


**Supplementary Figure 6:** Ancestry plot vizualizing inferred ancestory proportion on the y-axis per individual (x-axis) for K=8 and grouped by population with one row for each clustering method (STRUCTURE, InStruct, TESS3, and sNMF). The legend is standard across all methods and individuals are displayed in the same order. Clustering assignments were manually assigned to match population clusters across methods as closely as possible. However, groupings rather than colors should be compared across methods.

**
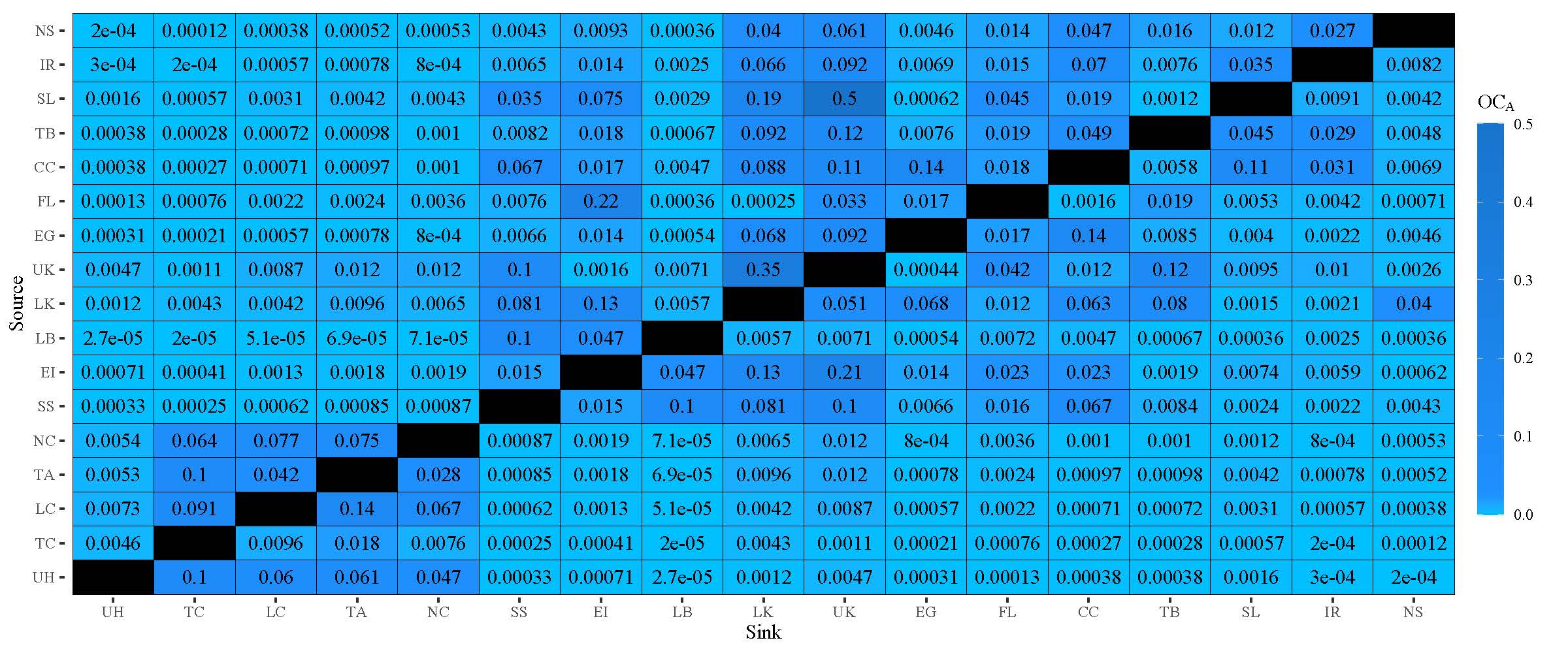
Supplementary Figure 7:** Heatmap with source population on the y-axis and sink population on the x-axis filled with asymmetric ocean current connectivity estimates (OC_A_; square rooted for normality) and shaded blue to indicate magnitudes of connectivity (darker blue = more connectivity).
